# The Appropriate Utilization of Beta-Blockers in Acute Coronary Syndrome Patients: An Improvement Initiative

**DOI:** 10.7759/cureus.74957

**Published:** 2024-12-02

**Authors:** Binayendu Prakash, Reeta R Mohanta, Santosh Kumar, Mandar M Shah

**Affiliations:** 1 Cardiology, Tata Main Hospital, Jamshedpur, IND

**Keywords:** : acute coronary syndrome, acute non-st elevation myocardial infarction, acute st-elevation myocardial infarction, beta-blockers, unstable angina

## Abstract

Background and objective

Beta-blockers are a cornerstone in the management of acute coronary syndrome (ACS), effectively reducing myocardial oxygen demand, preventing recurrent ischemia, and lowering the risk of arrhythmias and reinfarction. Despite several established guidelines, such as those by the American College of Cardiology/American Heart Association (ACC/AHA), advocating their use within 24 hours for eligible patients, beta-blockers remain underutilized in clinical practice. This study aimed to analyze beta-blocker utilization patterns in ACS management and evaluate the impact of targeted improvement initiatives on their appropriate use in eligible ACS patients.

Methods

A two-phase study was conducted at Tata Main Hospital, Jamshedpur, India. Phase I (October 2019 to May 2021) retrospectively analyzed the records of 1100 ACS patients. Phase II (July 2021 to October 2023) evaluated post-implementation outcomes of improvement measures, including physician education and a standardized prescription checklist. Patients were categorized based on adherence to beta-blocker guidelines and analyzed for utilization gaps. Statistical analysis was conducted using SPSS Statistics version 21 (IBM Corp., Armonk, NY), with significance set at a p-value <0.05.

Results

Post-intervention, beta-blocker adherence significantly improved from 74.5% to 92.7% (p<0.001). Late initiation without valid reasons (Group B) decreased from 15.5 to 3.6%, and non-initiation despite eligibility (Group C) decreased from 10 to 3.6%. These changes were statistically significant, highlighting the effectiveness of the improvement initiatives. Barriers to beta-blocker use included physician hesitancy when treating patients with relative contraindications. Targeted interventions, such as education and protocol standardization, helped to bridge this gap, aligning practices with global guideline recommendations.

Conclusions

Early and appropriate beta-blocker use in ACS management is crucial for improving patient outcomes. This study demonstrates that targeted interventions can significantly enhance guideline adherence, optimizing ACS care in resource-limited settings.

## Introduction

Acute coronary syndrome (ACS) refers to a spectrum of conditions caused by acute myocardial ischemia, commonly due to the rupture of an atherosclerotic plaque with subsequent thrombus formation. It is classified into ST-elevation myocardial infarction (STEMI), non-ST-elevation myocardial infarction (NSTEMI), and unstable angina, based on electrocardiographic changes and cardiac biomarkers [1,2]. ACS is associated with significant morbidity and mortality; in 2021, it accounted for 9.44 million deaths worldwide.

The management of ACS includes antiplatelets, anticoagulants, statins, and beta-blockers, alongside coronary revascularization when indicated [3,4]. While antiplatelets and statins are administered as loading doses on arrival, beta-blockers remain underutilized despite their proven benefits, primarily due to a lack of understanding related to their benefit in the ACS setting among treating physicians as well as the hesitancy to initiate them if any initial contraindication exists. 

Beta-blockers are crucial in reducing myocardial oxygen demand by lowering heart rate, blood pressure, and myocardial contractility. This reduction alleviates angina, prevents recurrent ischemia, and decreases the risk of ventricular arrhythmias and reinfarction [5,6]. It ultimately leads to a reduction in morbidity, rehospitalization, and mortality rates. Despite well-established guidelines, including the ones by the American College of Cardiology/American Heart Association (ACC/AHA), advocating for the initiation of beta-blockers within 24 hours in ACS patients without contraindications, underutilization persists. Contraindications include acute heart failure, hypotension, bradycardia, high-degree atrioventricular block, or active bronchospasm [7]. If contraindicated initially, patient eligibility should be reassessed within 24 hours [8]. Initial contraindications like heart failure and hypotension should be managed actively. An alternative to beta-blockers, such as ivabradine, can be used during such periods.

In a developing country like India with limited resources, not all patients with ACS can undergo revascularization. Hence, it is necessary to increase beta-blocker utilization to improve the clinical outcomes of ACS patients, and to achieve a reduction in length of stay and re-hospitalization, thereby improving the quality of life in the long term. To address this gap of underutilization, we conducted this study to analyze the usage patterns of beta-blockers in ACS management. Through targeted improvement initiatives, significant increases in beta-blocker utilization were achieved, leading to great benefits for ACS patients.

## Materials and methods

Study setting

The study was conducted at Tata Main Hospital, Jamshedpur, India: a 980-bedded tertiary care, multi-specialty hospital that caters to patients of all specialties. It is equipped with a cardiac intensive care unit (CICU) dedicated to managing ACS patients in the initial phase before being transferred to the cardiac ward. The hospital provides comprehensive cardiac care, including emergency services, coronary interventions, and long-term cardiac rehabilitation. It is equipped with a cath lab where approximately 1200 angiographies and 700 angioplasties are performed annually.

Study design

The study was conceived as a clinical audit and carried out in two phases. In phase I, a retrospective analysis of 1,100 medical records of patients admitted with ACS between October 2019 to May 2021 was performed. This phase identified patterns of beta-blocker utilization and gaps in adherence to established guidelines. In Phase II, following the implementation of targeted improvement initiatives, 1,100 subsequent ACS patient records were prospectively analyzed to assess the impact of the interventions between July 2021 to October 2023. Data were collected from consecutive cases to minimize selection bias.

Inclusion and exclusion criteria

The inclusion criteria were as follows: patients diagnosed with STEMI and NSTEMI based on clinical presentation, electrocardiographic changes, and biomarker profiles. Patients diagnosed with stable or unstable angina were excluded as consistency of diagnosis was not established in all such patients. Patients with ACS who had been referred late from other departments were also excluded from the study, as well as patients with incomplete medical records.

Data collection

The data from patients, either STEMI or NSTEMI, were pooled. Patients were divided into three groups based on the utilization pattern of beta-blockers. Group A consisted of patients receiving beta-blockers as per guidelines, either initiated within 24 hours of admission or started after 24 hours due to documented contraindications, or not administered due to clear contraindications as stated by ACC/AHA guidelines. Group B comprised patients receiving beta-blockers late (beyond 24 hours) without valid reasons, and Group C included patients not receiving beta-blockers despite an absence of contraindications.

The classification of patients into groups as per the beta-blocker utilization pattern is detailed in Table 1.

**Table 1:** Grouping of patients

Beta-blocker groups	
Group A	Beta-blockers started within 24 hours. Beta-blockers started after 24 hours due to valid reasons. Beta-blockers not given due to valid contraindications
Group B	Beta-blockers started late without valid reasons
Group C	Beta-blockers not started despite no contraindications

Ideally, all patients should be in Group A, and no patients should be in Group B or C. The reason for delayed or no initiation of beta-blocker was analyzed. Key interventions implemented to address identified gaps included educational programs and regular training sessions for physicians and nurses focusing on the benefits and contraindications of beta-blockers in ACS. A standardized protocol was Introduced in the form of a checklist for evaluating beta-blocker eligibility and initiation early during ACS management. This checklist was attached with the case files of all eligible patients; it mentioned contraindications to beta-blockers to be checked on a daily basis. Initial resistance to compliance was overcome by regular auditing and feedback on a weekly basis to enhance compliance.

Statistical analysis

Statistical analysis was performed to check the significance of improvement. Data were collected using MS Excel. Patients with missing data were excluded beforehand. Continuous variables were expressed as means ± standard deviation (SD) and ranges; categorical variables were expressed as numbers and percentages. Differences between the two groups were assessed by the chi-square test or Fisher's exact test for categorical variables and the Mann-Whitney U test for continuous variables. All tests were two-sided, and a p-value <0.05 was considered statistically significant. The analysis was performed using SPSS Statistics version 21 (IBM Corp., Armonk, NY).

## Results

A total of 2,200 patients (1,100 pre-intervention, 1,100 post-intervention) were included in the study. The mean age of patients was 62 years (range: 28-89 years); 24.5% of patients were female and 75.5% were male. Both groups had statistically comparable demographic profiles. The data related to categorical variables in both groups are presented in Table 2.

**Table 2:** Categorical variables

Variables (n=2200)		N (%)
Age, years	≤40	110 (5.0%)
	41-50	230 (10.5%)
	51-60	550 (25.0%)
	61-70	770 (35.0%)
	>70	540 (24.5%)
Gender	Female	540 (24.5%)
	Male	1660 (75.5%)
Beta-blocker groups	Group A	1840 (83.6%)
	Group B	210 (9.5%)
	Group C	150 (6.8%)
Beta-blocker medicine	Pre-beta-blocker	1100 (50.0%)
	Post-beta-blocker	1100 (50.0%)

The test statistics (χ²=3.55 and p=0.471) in age distribution for pre/post-intervention groups indicate no statistically significant difference. Any observed differences may be due to chance; likewise, the test statistics of χ²=1.68 and p=0.639 also suggested no statistically significant difference in gender distribution between the pre-and post-intervention groups. A retrospective analysis of 1100 patients showed that 74.5% were in Group A, 15.5% in Group B, and the remaining 10% were in Group C.

After the implementation of improvement measures, further analysis showed that the number of patients in Group A increased from 820 (74.5%) to 1020 (92.7%), which was statistically significant, indicating that more deserving patients received beta-blockers on time. It also indicated the effectiveness of improvement initiatives that were implemented, such as checklists and regular awareness sessions for treating physicians and nurses. In the younger age group (<40 years), the utilization improved much more, although no notable reason could be found. In Group B, the number of patients decreased from 170 (15.5%) to 40 (3.6%); in Group C, the number of patients decreased from 110 (10%) to 40 (3.6%). These results do not align with the study objective of increasing the number of patients in Group A and reducing the number of patients in both Group B and Group C. The key findings are shown in Table 3.

**Table 3:** Key findings from patient groups

Variables (n=2200)		(N=2200)		Test statistics	P-value	
		Pre-beta-blocker medicine (n=1100)	Post-beta-blocker medicine (n=1100)			
		N (%)	N (%)			
Age, years	≤40	30 (2.7%)	80 (7.3%)	χ²=3.55	0.471	
	41-50	120 (10.9%)	110 (10.0%)			
	51-60	260 (23.6%)	290 (26.4%)			
	61-70	430 (39.1%)	340 (30.9%)			
	>70	260 (23.6%)	280 (25.5%)			
Gender	Female	250 (22.7%)	290 (26.4%)	χ²=1.68	0.639	
	Male	850 (77.3%)	810 (73.6%)			
Beta-blocker groups	Group A	820 (74.5%)	1020 (92.7%)	χ²=36.86	0.001	
	Group B	170 (15.5%)	40 (3.6%)			
	Group C	110 (10.0%)	40 (3.6%)			

The test statistics χ²=36.86 and p=0.001 indicated a statistically significant increase in patients who received beta-blockers on time, thereby improving outcomes of ACS management. This suggests that the observed differences are not due to random chance but rather reflect a meaningful and measurable shift towards betterment. This significant change highlights the potential effectiveness of the intervention, underscoring its positive impact on the population under study.

## Discussion

The role of beta-blockers in ACS is multifaceted. They reduce myocardial oxygen demand by lowering heart rate and blood pressure while mitigating contractility [9]. These effects improve clinical outcomes by reducing angina, ischemia, ventricular arrhythmias, and reinfarction rates. Additionally, beta-blockers may prevent sudden cardiac death by stabilizing arrhythmic thresholds [10]. In STEMI patients, beta-blockers provide additional benefits, including reducing reperfusion arrhythmias during thrombolysis or percutaneous coronary intervention [11,12].

This study demonstrated a significant improvement in beta-blocker utilization post-intervention, with Group A patients increasing from 74.5% to 92.7%. These results highlight the effectiveness of targeted initiatives in enhancing guideline adherence. The decrease in inappropriate delays (Group B) and omissions (Group C) further emphasizes the value of standardized protocols and education. These positive results will lead to better patient outcomes in the form of reduced morbidity, rehospitalization, and mortality in the long term.

Barriers to beta-blocker use included physician hesitancy when encountering patients with relative contraindications, such as transient hypotension or mild heart failure. Interventions focused on addressing misconceptions and emphasizing reassessment protocols significantly improved compliance. For example, hypotension due to hypovolemia or medication effects was managed through optimization rather than excluding beta-blockers. Educating physicians and nurses, as well as introducing checklists that acted as a ready reckoner to check for eligibility and contraindications of beta-blockers, addressed these gaps, as evidenced by the significant post-initiative improvement in adherence rates. 

The global utilization of beta-blockers in ACS varies, ranging from 47% to 81% in high-income countries, whereas rates in low- and middle-income countries like India are often lower: between 47% and 65% [8,13-15]. The improvement observed in this study highlights the potential for targeted interventions to close this gap. It is of pivotal importance to optimize drugs in ACS patients in low- and middle-income countries as not all patients can afford to undergo revascularization of affected coronary arteries due to financial constraints and limited resources. 

This study was conducted at a single center. Expanding these initiatives to multiple centers, incorporating multidisciplinary approaches, and exploring long-term outcomes, such as mortality reduction and rehospitalization rates, would further strengthen the evidence base. The positive outcome from guided interventions like checklists in terms of improving adherence to the protocol can be extrapolated to other hospitals and healthcare centers on a large scale, thereby improving the management of ACS patients as well as the overall health status of the general population.

## Conclusions

Beta-blockers are a cornerstone in the management of ACS, offering immediate relief and long-term protection against cardiac complications. Their early and consistent use, coupled with strategies to address barriers to implementation, can significantly enhance patient outcomes by limiting morbidity and mortality and improving the quality of life. By focusing on education, protocol development, and reassessment strategies, healthcare providers can ensure that more patients receive the life-saving benefits of beta-blocker therapy. Expansion of such initiatives across healthcare centers can improve outcomes for such patients. The findings and strategies discussed in this study can be implemented in various healthcare setups without imposing too much burden on resources in low- and middle-income countries to improve their health statistics. We recommend further studies to validate the clinical outcomes of interventions adopted in this study. In the future, innovative digital tools like mobile-based apps can be developed to increase adherence to treatment protocols.
